# Increased expression and altered localization of cathepsin Z are associated with progression to jaundice stage in primary biliary cholangitis

**DOI:** 10.1038/s41598-018-30146-w

**Published:** 2018-08-07

**Authors:** Yoshihiro Aiba, Kenichi Harada, Masahiro Ito, Takashi Suematsu, Shinichi Aishima, Yuki Hitomi, Nao Nishida, Minae Kawashima, Mitsuhisa Takatsuki, Susumu Eguchi, Shinji Shimoda, Hitomi Nakamura, Atsumasa Komori, Seigo Abiru, Shinya Nagaoka, Kiyoshi Migita, Hiroshi Yatsuhashi, Katsushi Tokunaga, Minoru Nakamura

**Affiliations:** 1 Clinical Research Center, National Hospital Organization Nagasaki Medical Center, Omura, Japan; 2 Department of Human Pathology, Kanazawa University Graduate School of Medicine, Kanazawa, Japan; 3 Department of Hepatology, Nagasaki University Graduate School of Biomedical Sciences, Omura, Nagasaki, Japan; 4 Central Electron Microscope Laboratory, Nagasaki University School of Medicine, Nagasaki, Nagasaki, Japan; 5 Departments of Pathology & Microbiology, Faculty of Medicine, Saga University, Saga, Japan; 6 Department of Human Genetics, Graduate School of Medicine, The University of Tokyo, Tokyo, Japan; 7 The Research Center for Hepatitis and Immunology, National Center for Global Health and Medicine, Ichikawa, Chiba, Japan; 8 Japan Science and Technology Agency (JST), Tokyo, Japan; 9 Department of Surgery, Nagasaki University Graduate School of Biomedical Sciences, Nagasaki, Japan; 10 Department of Medicine and Biosystemic Science Graduate School of Medical Sciences, Kyushu University, Fukuoka, Fukuoka, Japan; 11 Headquarters of PBC Research in the National Hospital Organization Study Group for Liver Disease in Japan (NHOSLJ), Omura, Japan

## Abstract

Our recent genome-wide association study found that the *NELFCD*/*CTSZ* locus was significantly associated with progression of primary biliary cholangitis (PBC) to jaundice stage in the Japanese population. In this study, we investigated the role of cathepsin Z in the etiology and pathology of PBC. Serum cathepsin Z levels were measured using enzyme-linked immunosorbent assay. The expression and localization of cathepsin Z in liver specimens were analyzed by western blotting and immunohistochemistry. In PBC patients, serum cathepsin Z levels were significantly increased with disease progression. In addition, its levels were positively correlated with alanine transaminase, aspartate transaminase and total bilirubin, and were negatively correlated with platelet count and albumin. Cathepsin Z expression was markedly increased in hepatocytes at later stages of PBC, and its localization was altered from the peri-bile canaliculus to the cytoplasm, where a fraction was no longer colocalized with endosomal/lysosomal vesicles. Similar altered expression of cathepsin Z was observed in end-stage of other cholestatic liver diseases including sepsis, obstructive jaundice, and Alagille syndrome. Our results indicate that altered expression and localization of cathepsin Z in hepatocytes are characteristic features of PBC and other cholestatic liver diseases, and are implicated in the progression of PBC.

## Introduction

Primary biliary cholangitis (PBC), previously referred to as primary biliary cirrhosis, is a chronic liver disease characterized by the destruction of intrahepatic bile ducts and progressive cholestasis, leading to liver cirrhosis, jaundice, and end-stage hepatic failure^1^. Although treatment with ursodeoxycholic acid (UDCA) alone or in combination with bezafibrate is very effective for normalizing liver function and preventing PBC progression^2,3^, approximately 10–30% of PBC patients are resistant to these treatments and progress to end-stage hepatic failure^4–6^. We previously reported that the pattern of PBC progression can be classified into three different types: (1) slowly progressive, which does not influence life expectancy; (2) progressive, which proceeds to liver cirrhosis and/or portal hypertension, but rarely to jaundice and end-stage hepatic failure; and (3) progressive, which proceeds to jaundice and end-stage hepatic failure^4^. Using this method of classification, we recently found that genetic polymorphisms (rs13720 and rs163800) at the *NELFCD*/*CTSZ* locus were associated with progression to jaundice stage in PBC in a Japanese population^7^.

Cathepsin Z is ubiquitously expressed in tissues and is highly expressed in the liver, spleen, lung, and kidney^8^. Cathepsin Z is a member of the cysteine cathepsins located in the lysosome, and exhibits exopeptidase activity. Cathepsin Z cleaves the carboxyl-terminus of beta integrin receptor, macrophage antigen-1, lymphocyte function-associated 1, and C-X-C motif chemokine 12, and regulates the adhesion, migration, and maturation of immune cells^9^. In addition, cathepsin Z cleaves extended polyglutamine tracts^10^, huntingtin^11^, and γ-enolase^12^, and is involved in the development of neurodegenerative diseases such as Alzheimer’s disease^13^ and Huntington’s disease^12^. Cathepsin Z expression is locally increased in cancer^14,15^ and *Helicobacter pylori*–associated gastritis^14^, and systemically increased in severe trauma^16^.

The expression and functional role of cathepsin Z have not been fully characterized in liver diseases, including PBC. In this study, we investigated pathoetiological roles of cathepsin Z in disease progression in PBC.

## Results

### Serum cathepsin Z levels increase with PBC progression

**Figure 1:**
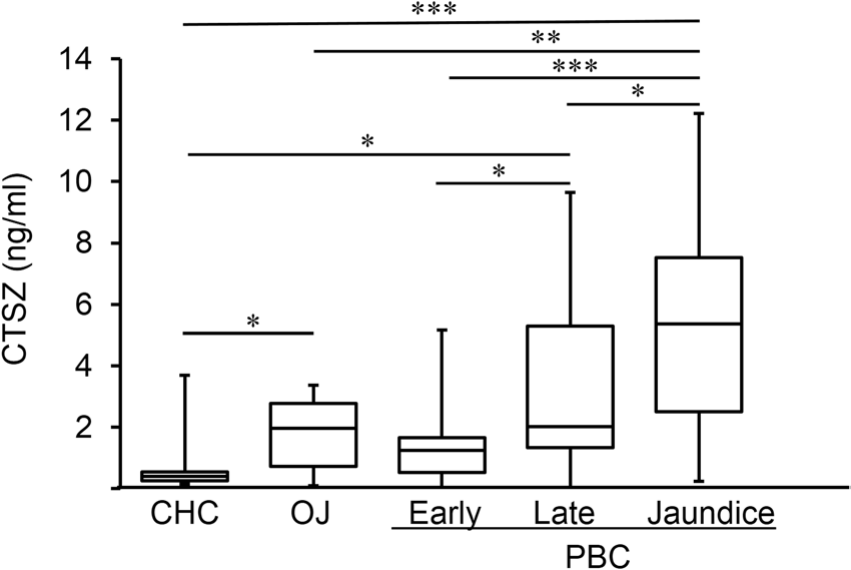
Serum cathepsin Z levels in liver diseases. (**A**) Serum cathepsin Z levels were significantly higher in jaundice-stage PBC (n = 29) than in CHC (n = 20) and early-stage (n = 25) and late-stage (n = 17) PBC. Levels in obstructive jaundice (OJ, n = 12) were significantly higher than in CHC. Statistical analysis was performed using the Mann-Whitney U-test; *P < 0.05, **P < 0.01, ***P < 0.001.

We measured serum cathepsin Z levels in three clinical stages of PBC (early-stage n = 25, late-stage n = 17, jaundice-stage n = 29), in two clinical stages of CHC (early-stage n = 10, late-stage n = 10), and in obstructive jaundice (n = 12) (Fig. 1). Serum cathepsin Z levels were significantly higher in late-stage PBC (2.03 ± 3.09 ng/ml) than in early-stage PBC (1.24 ± 1.26 ng/ml) (*P* = 0.03). Serum cathepsin Z levels were also significantly higher in jaundice-stage PBC (5.37 ± 3.46 ng/ml) as compared to early-stage PBC (*P* = 5.3 × 10^−7^) and late-stage PBC (*P* = 0.04). In contrast to these results, there was no significant difference in serum cathepsin Z levels between early-stage CHC and late-stage CHC (data not shown), and levels were significantly lower in CHC (0.80 ± 1.07 ng/ml) than in late-stage and jaundice-stage PBC (*P* = 0.04 and *P* = 2.37 × 10^−6^, respectively). (Fig. 1). Serum cathepsin Z levels in obstructive jaundice (n = 12, 1.78 ± 1.15 ng/ml) were significantly higher compared to those of CHC (*P* = 0.02), but significantly lower than those of jaundice-stage PBC (*P* = 0.002).

**Figure 2:**
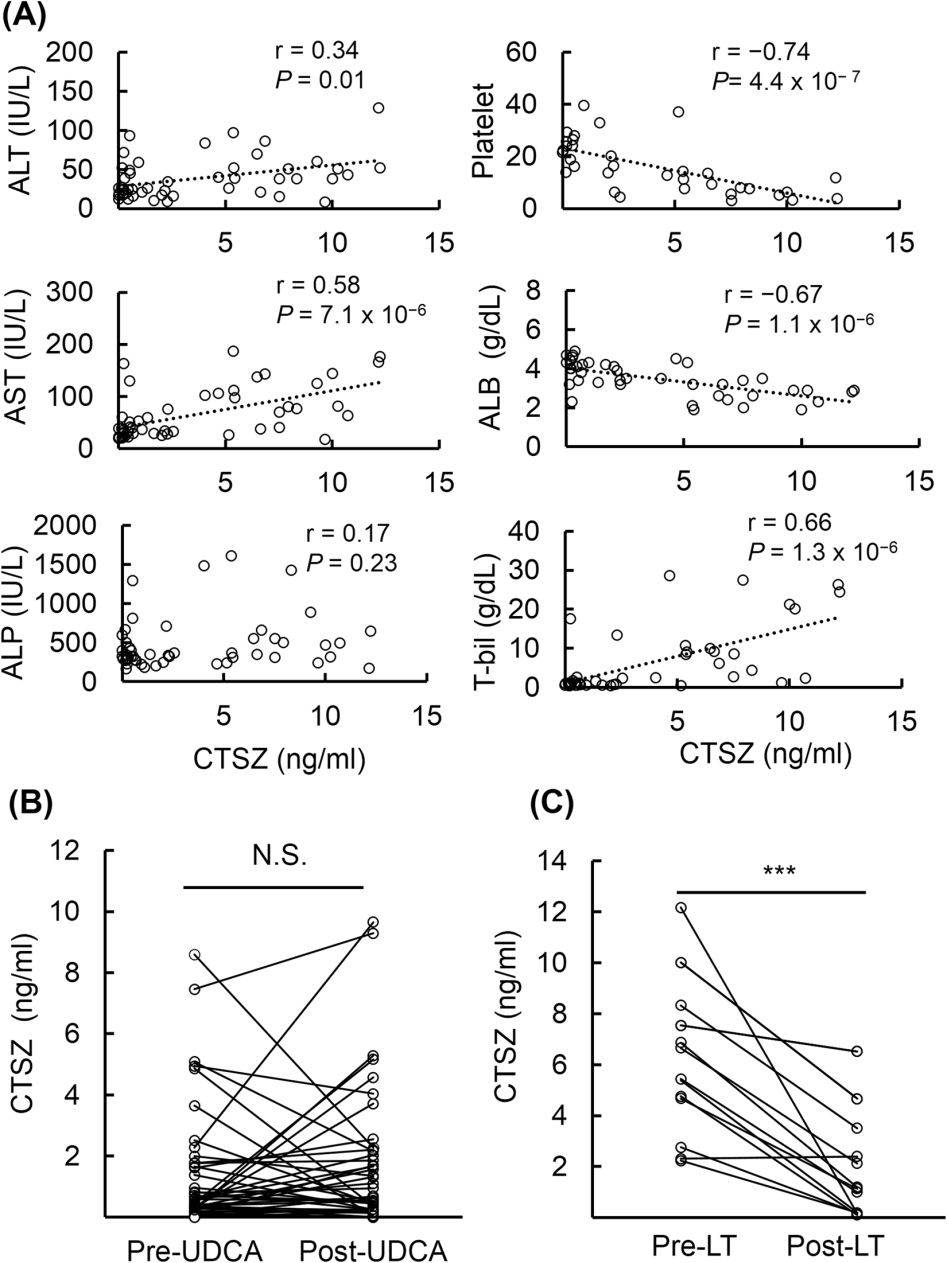
Association of serum cathepsin Z levels with biochemical markers, UDCA treatment, and liver transplantation in PBC. (**A**) Correlations between levels of cathepsin Z and biochemical markers in PBC patients (n = 71) were assessed using Spearman’s correlation coefficient and *P*-value. Serum cathepsin Z levels were positively correlated with ALT, AST, and total bilirubin, and were negatively correlated with platelet count and ALB in PBC. Serum cathepsin Z levels did not change after UDCA treatment (n = 43) (**B**), but levels were significantly decreased after liver transplantation (n = 13) (**C**). Statistical analysis was performed using the Mann-Whitney U-test; ***P < 0.001. ALT, alanine transaminase; AST, aspartate transaminase; ALP, alkaline phosphatase; ALB, albumin; UDCA, ursodeoxycholic acid; LT, liver transplantation; N.S., not significant.

We next investigated whether cathepsin Z levels correlated with other biochemical markers of liver function in PBC patients (Fig. 2A). Serum cathepsin Z levels were positively correlated with alanine transaminase (r = 0.34, *P* = 0.01), aspartate aminotransferase (r = 0.58, *P* = 7.1 × 10^−6^) and total bilirubin (r = 0.66, *P = *1.3 × 10^−6^), and were negatively correlated with platelet count (r = −0.74, *P* = 4.4 × 10^−7^) and albumin (r = −0.67, *P* = 1.1 × 10^−6^). On the other hand, its levels were not significantly correlated with alkaline phosphatase.

We investigated whether UDCA treatment and liver transplantation affect serum cathepsin Z levels. During UDCA treatment, 4 out of 33 PBC patients at the early-stage progressed to late-stage (n = 3) or jaundice-stage (n = 1), and 1 out of 7 at late-stage PBC patients progressed to jaundice-stage. Serum cathepsin Z levels were not significantly decreased after UDCA treatment (n = 43, 0.58 ± 2.01 ng/ml pre-UDCA vs. 0.66 ± 2.26 ng/ml post-UDCA, *P* = 0.63; period of UDCA treatment was 52.3 ± 41.1 months) (Fig. 2B). Liver transplantation, however, significantly decreased serum cathepsin Z levels (n = 13, *P* = 8.1 × 10^−4^). The serum cathepsin Z concentration of jaundice-stage PBC patients was measured before transplantation (6.48 ± 2.77 ng/ml) and after transplantation (1.18 ± 2.32 ng/ml), with a post-transplant observation period of 16.7 ± 14.8 months (Fig. 2C). These results suggest that serum cathepsin Z is a surrogate marker for severe progression with cholestasis in PBC.

### Advanced PBC is associated with increased cathepsin Z protein levels in the liver

**Figure 3:**
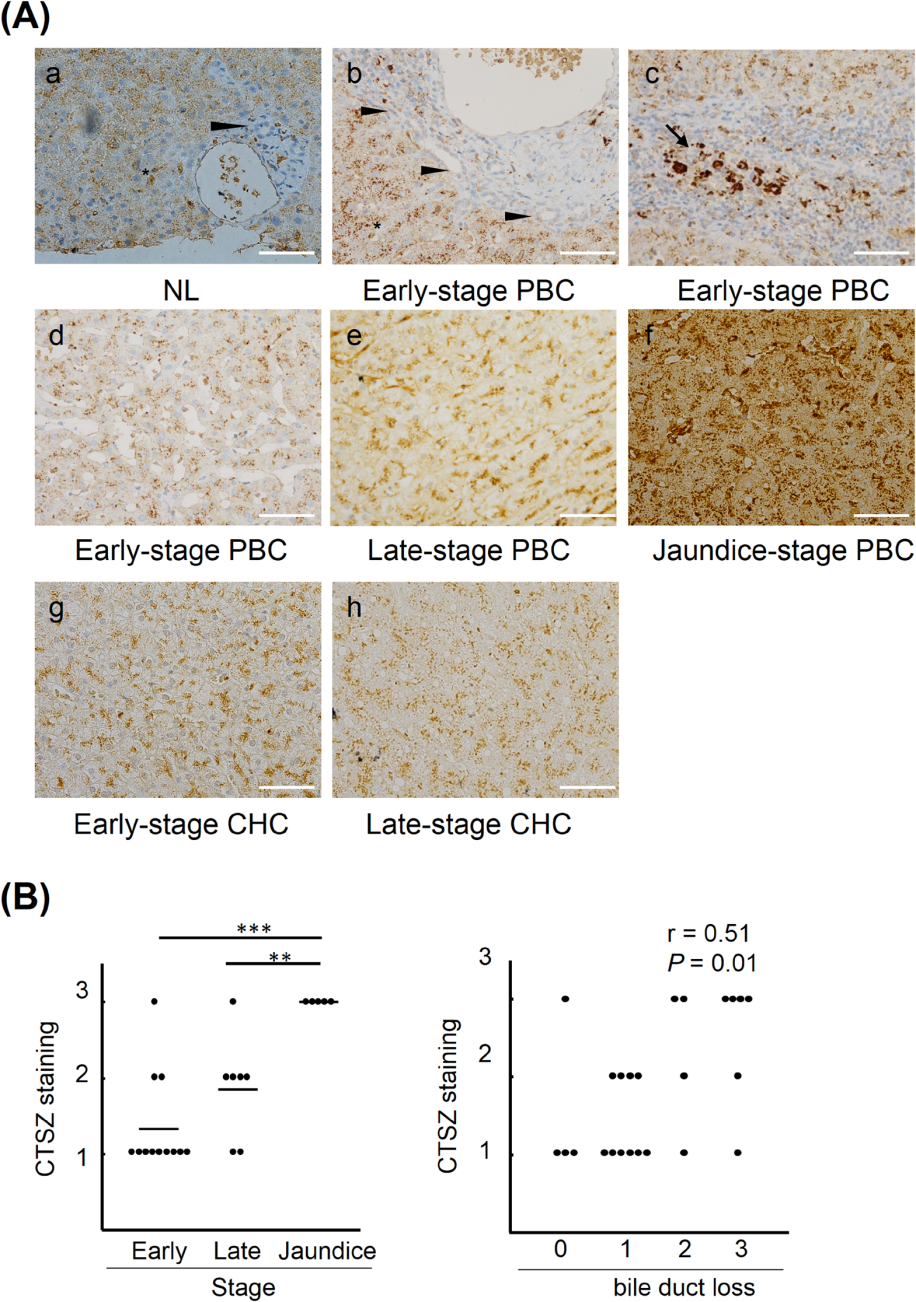
Immunohistochemical staining of cathepsin Z in liver tissue. (**A**) Liver specimens from non-diseased subjects (NL); early-, late-, and jaundice-stage PBC; and CHC were stained using an antibody to cathepsin Z (×40). In the NL samples (**a**) and specimens from patients with early-stage PBC (b,c), cathepsin Z was strongly expressed in hepatocytes, Kupffer cells (asterisk), endothelial cells, and some infiltrating mononuclear cells, in particular monocytes and macrophages (arrow), and was weakly expressed in bile duct (arrowhead). The expression of cathepsin Z in hepatocytes was markedly increased with progression of PBC (d e,f) but not CHC (g,h). Representative images are shown. Scale bar: 100 μm. (**B**) The intensity of cathepsin Z staining in hepatocyte of PBC liver (n = 24; early-stage n = 12, late-stage n = 7, jaundice-stage n = 5) were scored based on the flowing scale: 0, negative staining; 1, weak staining; 2, moderate staining; 3, strong staining. The intesnsity of cathepsin Z staining was significantly increased with progression of PBC (left) (average score of cathepsin Z staining in early-stage; 1.33, late-stage; 1.85, jaundice-stage; 3.00). Horizontal lines represent average values for each group. Statistical analysis was performed using Student’s t-test; **P < 0.01, ***P < 0.001. In addition, the intensity of cathepsin Z staining was positively correlated with bile duct loss in PBC liver (right). The correlation was assessed using Spearman’s correlation coefficient and *P*-value.

To investigate the role of cathepsin Z in progression of PBC, immunohistochemical staining of cathepsin Z was performed in liver tissues (Fig. 3). Cathepsin Z was expressed in hepatocytes, Kupffer cells, endothelial cells, and a small portion of infiltrating mononuclear cells, mainly in macrophages and monocytes, in both non-diseased liver (NL) and early-stage PBC, whereas cathepsin Z was minimally expressed in bile ducts (Fig. 3Aa–c). Interestingly, the intensity of cathepsin Z staining in hepatocytes increased with progression of PBC (Fig. 3Ad–f), which was also shown by immunohistochemical scoring (jaundice-stage v.s early-stage, *P* = 4.9 × 10^−5^, jaundice-stage v.s late-stage, *P* = 4.7 × 10^−3^) (Fig. 3B). In addition, the intensity of cathepsin Z staining was positively correlated with bile duct loss in PBC (r = 0.51, *P* = 0.01) (Fig. 3B). By contrast, the intensity of cathepsin Z staining was not increased with progression of CHC (Fig. 3Ag,h).

### Cathepsin Z localization is altered in jaundice-stage PBC

**Figure 4:**
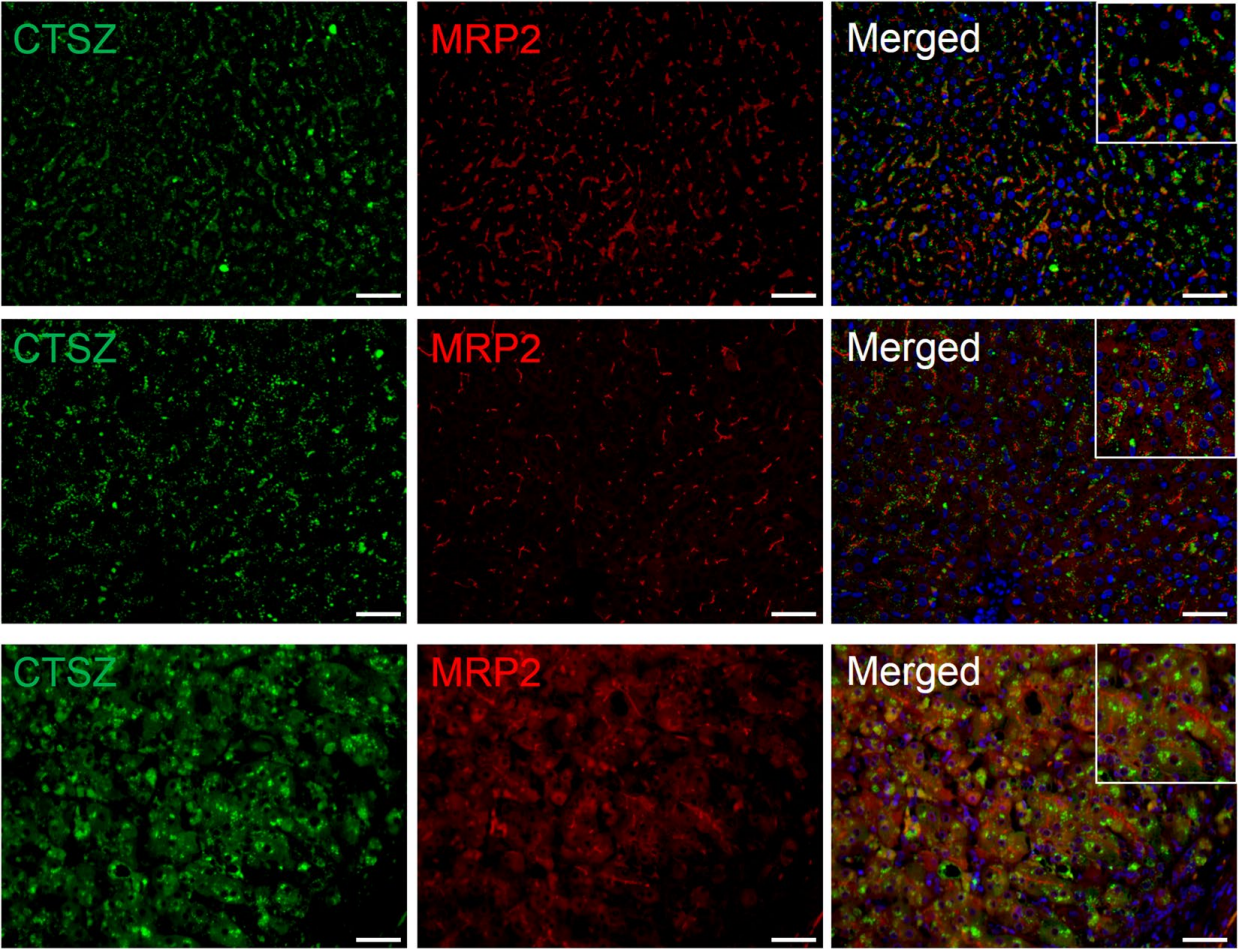
Simultaneous immunofluorescence staining of cathepsin Z and MRP2 in hepatocytes. Liver specimens from non-diseased subjects (NL) and early- or jaundice-stage PBC were simultaneously stained with antibodies to cathepsin Z and bile canalicular marker MRP2 (×20). Nuclei were stained with DAPI (bule). Cathepsin Z (CTSZ; green) was observed proximal to bile canalicular marker MRP2 (red) in hepatocytes of NL (upper), whereas it was observed in the cytoplasm of hepatocytes from patients with early- (middle) and jaundice-stage PBC (lower). Scale bar: 50 μm.

**Figure 5:**
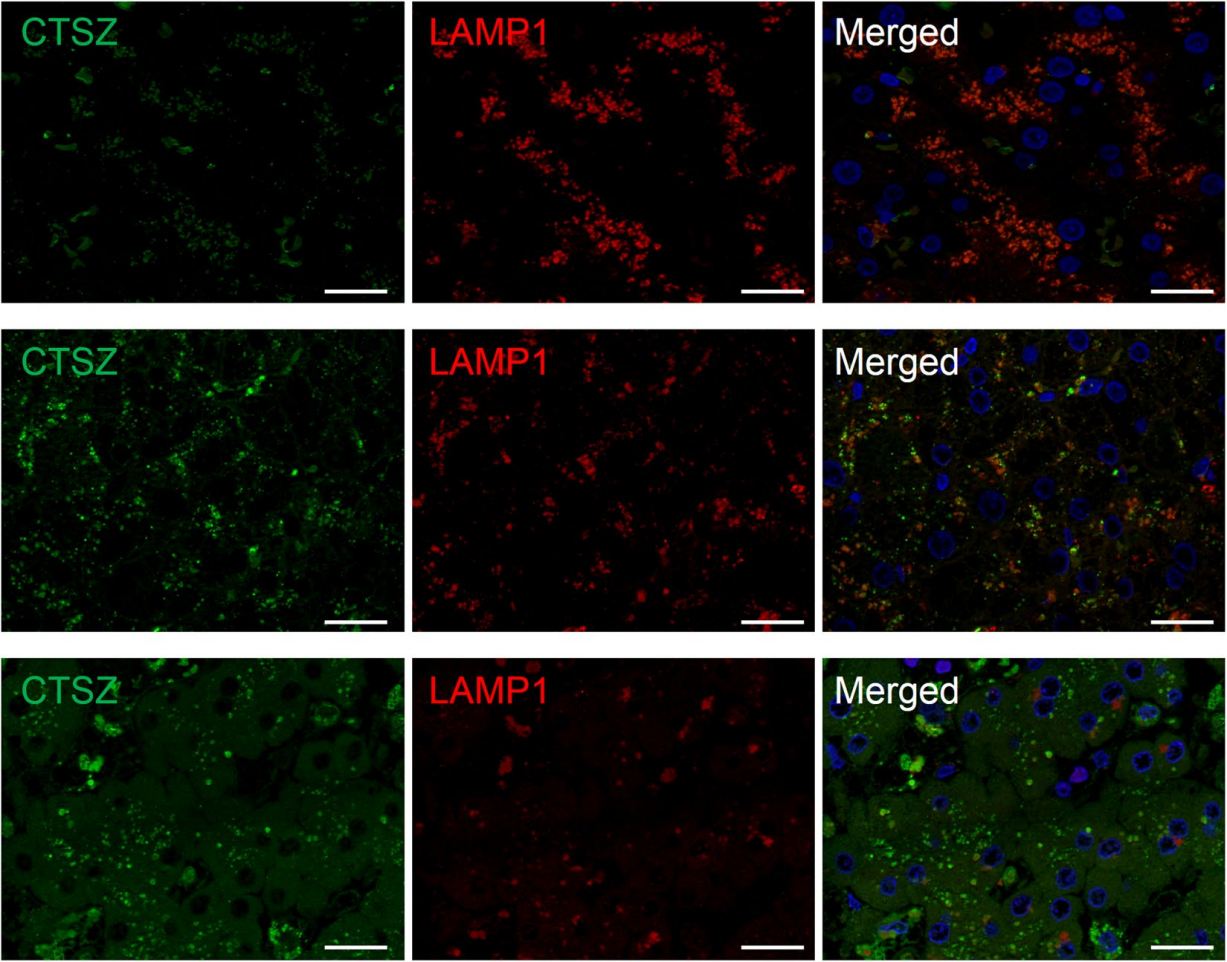
Simultaneous immunofluorescence staining of cathepsin Z and LAMP1 in hepatocytes. Liver specimens from non-diseased subjects (NL) and early- or jaundice-stage PBC were simultaneously stained with antibodies to cathepsin Z and lysosomal marker LAMP1 (×63). Nuclei were stained with DAPI (bule). Colocalization of cathepsin Z (CTSZ; green) with LAMP1 (red) was observed in hepatocytes of NL (upper) and early-stage PBC (middle), whereas colocalization was rare in hepatocytes of jaundice-stage PBC (lower). Scale bar: 25 μm.

We analyzed the intracellular distribution of cathepsin Z in the liver by immunofluorescence staining using bile canalicular marker MRP2 and lysosomal marker LAMP1. Cathepsin Z was predominantly localized adjacent to MRP2 in hepatocytes of NL (Fig. 4 upper) and in early-stage PBC (Fig. 4 middle). In jaundice-stage PBC, cathepsin Z was predominantly localized in the cytoplasm of hepatocytes, and the localization of MRP2 shifted from the bile canaliculus to the cytoplasm (Fig. 4 lower). Cathepsin Z was colocalized with LAMP1 in hepatocytes of NL (Fig. 5 upper) and in early-stage PBC (Fig. 5 middle), whereas colocalization was rarely observed in jaundice-stage PBC (Fig. 5 lower). These results suggest that the cathepsin Z is translocated from lysosomes near the bile canaliculus to the cytoplasm in jaundice-stage PBC.

### Increased cathepsin Z protein in jaundice-stage PBC is due to non-transcriptional regulation

**Figure 6:**
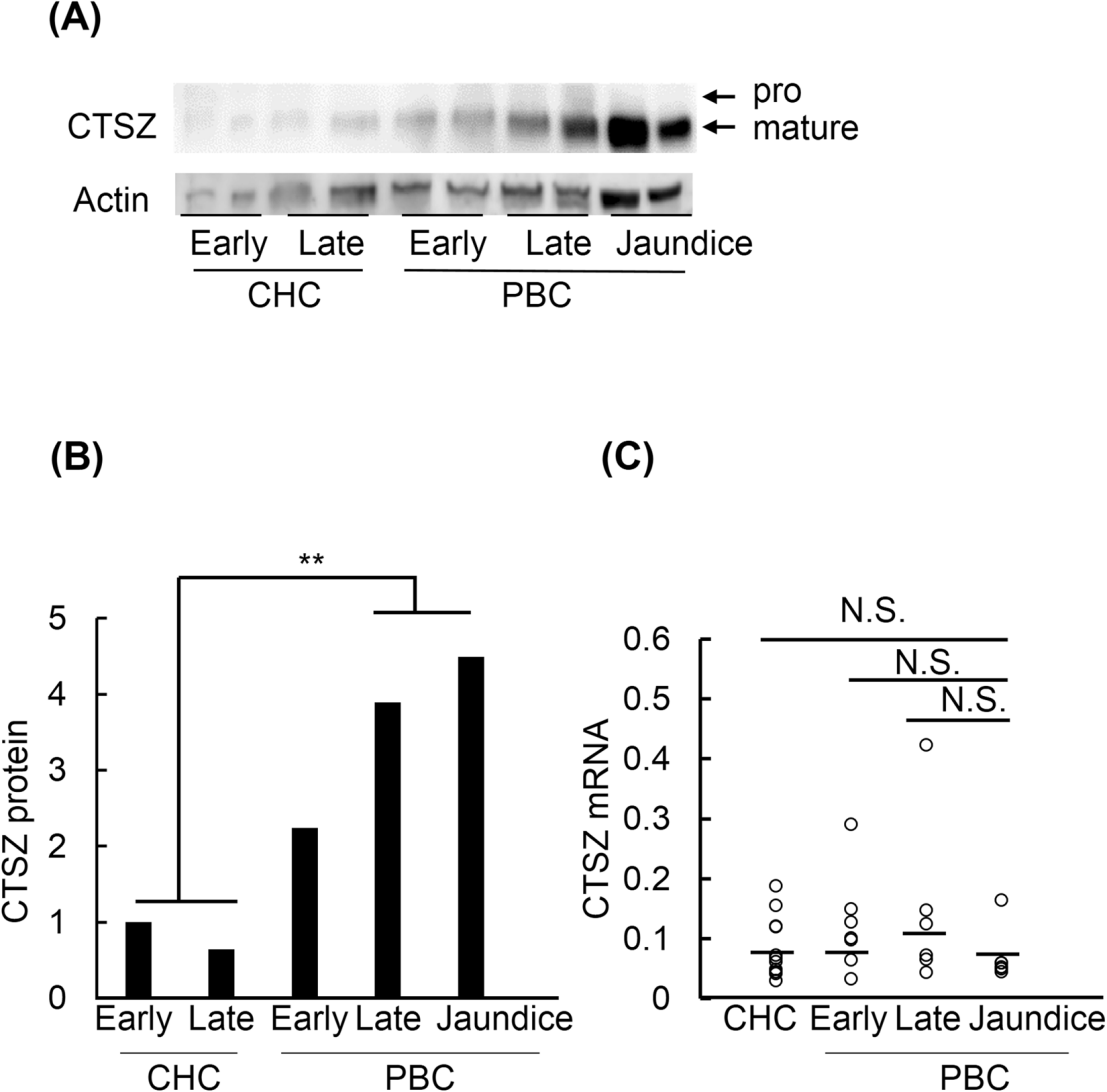
Expression of cathepsin Z in liver tissues. (**A**) Western blotting of cathepsin Z using homogenate from liver tissues of CHC (early-stage n = 2, late-stage n = 2) and PBC (early-stage n = 2, late-stage n = 2, jaundice-stage n = 2). Actin was used as an internal control. The original full-length blot images are shown in Supplementary Fig. 1. (**B**) Densitometric analysis showed that cathepsin Z (mature form) was significantly increased at later stages of PBC as compared to CHC. Statistical analysis was performed using Welch’s t-test. (**C**) The expression of cathepsin Z mRNA relative to GAPDH in liver tissues of CHC (n = 11) and PBC (early-stage n = 7, late-stage n = 6, jaundice-stage n = 6) is shown. There were no significant differences (N.S.) in the expression of cathepsin Z mRNA between CHC and different stages of PBC. Statistical analysis was performed using Welch’s t-test. Horizontal lines represent median values.

Cathepsin Z is synthesized as a preproenzyme and is processed to a mature form in the lysosome. To investigate which form of cathepsin Z is increased with progression of PBC, western blotting was performed on liver tissues. Western blotting showed that the mature form of cathepsin Z protein was markedly increased at later stage of PBC as compared to CHC (Fig. 6A), which was also confirmed by densitometric analysis (*P* = 3.7 × 10^−3^) (Fig. 6B). Under our experimental conditions, the pro-form of cathepsin Z was barely detected in both PBC and CHC samples (Fig. 6A). These results suggest that maturation of cathepsin Z occurs normally in the livers of jaundice-stage PBC patients.

To investigate whether the increase in cathepsin Z protein was due to transcriptional regulation, transcript levels of cathepsin Z were measured in liver samples (Fig. 6C). There were no significant differences in transcript levels between CHC and the different stages of PBC, suggesting that the increased cathepsin Z protein level observed in jaundice-stage PBC is not due to transcriptional regulation.

### Increased expression and altered localization of cathepsin Z are observed in other cholestatic liver diseases

**Figure 7:**
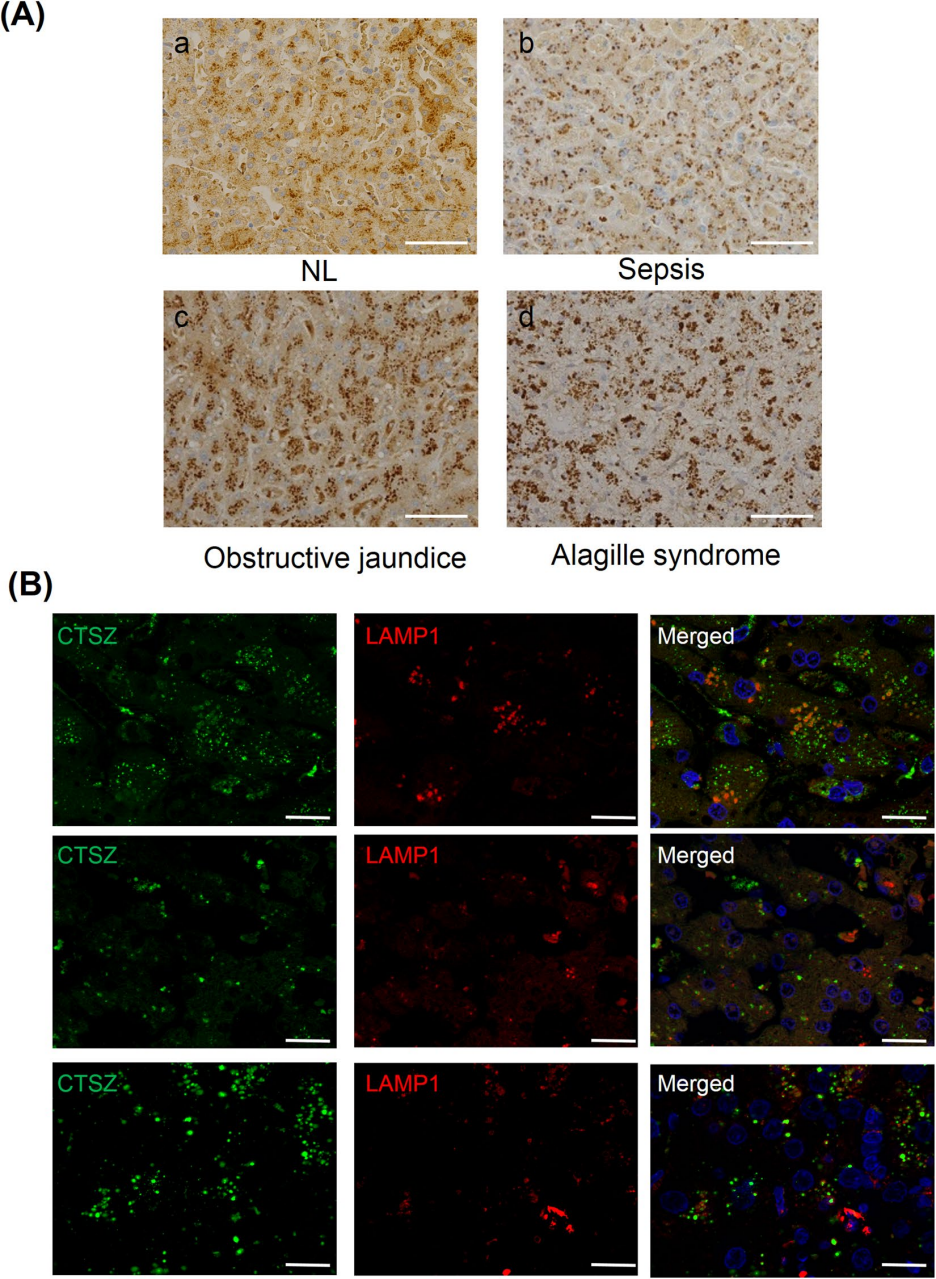
Expression and localization of cathepsin Z in cholestatic liver diseases. (**A**) Immunohistochemical staining for cathepsin Z in liver specimens from non-diseased subjects (NL; a) and patients with cholestatic liver diseases including sepsis (b), Alagille syndrome (c), and obstructive jaundice (d) (×40). The expression of cathepsin Z was increased in hepatocytes of patients with cholestatic liver diseases, and its localization was altered from the bile canaliculus to the cytoplasm. (**B**) Simultaneous immunofluorescence staining of cathepsin Z and LAMP1 in liver specimens. (×63). Nuclei were stained with DAPI (bule). Colocalization of cathepsin Z (CTSZ; green) with LAMP1 (red) was rarely found in liver specimens from patients with sepsis (upper), obstructive jaundice (middle), and Alagille syndrome (lower). Scale bar: 25 μm.

To investigate whether increased expression and altered localization of cathepsin Z is associated with cholestasis, the expression and localization of cathepsin Z was analyzed at the end stage of other cholestatic liver diseases. Cathepsin Z protein expression was markedly increased in hepatocytes at the end stage of sepsis, Alagille syndrome, and obstructive jaundice as compared to NL (Fig. 7A). In addition, the cathepsin Z was translocated from the bile canaliculus region of hepatocytes to the cytoplasm, particular in sepsis and Alagille syndrome (Fig. 7A) and immunofluorescence staining showed that cathepsin Z was no longer colocalized with LAMP1 in all three diseases (Fig. 7B). These results suggest that increased expression of cathepsin Z with altered localization in hepatocytes is a common phenomenon in end-stage of cholestatic liver diseases.

## Discussion

In this study, we report several novel findings about the role of cathepsin Z in the pathogenesis of cholestatic liver diseases, particularly primary biliary cholangitis. First, Serum cathepsin Z was significantly increased with the progression of PBC. Second, its levels were significantly correlated with several biochemical markers for liver function including alanine transaminase, aspartate aminotransferase, platelet count, total bilirubin, and albumin. Third, cathepsin Z was markedly increased in hepatocytes from patients with later stages of PBC. Forth, in hepatocytes of patients with jaundice-stage PBC, cathepsin Z was translocated from the peri-bile canalicular membrane region to the cytoplasmic region, where it no longer colocalized with lysosomal marker LAMP1. Fifth, increased expression and altered localization of cathepsin Z were also observed in hepatocytes at the end stage of other cholestatic liver diseases, including Alagille syndrome, obstructive jaundice, and sepsis. Finally, in contrast to PBC and other cholestatic liver diseases, cathepsin Z was not increased in sea and liver tissues of CHC. Our results indicate that altered expression and localization of cathepsin Z is a phenomenon particular to cholestatic liver diseases such as PBC and is implicated in the progression of PBC.

Increased serum cathepsin Z levels were reported in non-survivors after trauma^16^ and in cancer patients with shorter overall survival^17^, indicating that cathepsin Z is a potential serum marker for inflammation or chemosensitivity. In this study, increased serum cathepsin Z levels with PBC progression were significantly correlated with several biochemical markers for liver function (platelet count, albumin, total bilirubin, alanine transaminase, and aspartate aminotransferase) and were normalized after liver transplantation. On the other hand, serum cathepsin Z levels were significantly lower in CHC than in PBC and obstructive jaundice. In addition, its levels were not correlated with alkaline phosphatase which is a major diagnostic maker of bile duct injury and cholestasis in PBC. These results indicate that cathepsin Z is a potential serum marker for severe progression with cholestasis in PBC.

The lysosomal enzymes in hepatocytes are mainly excreted from the bile canaliculus via lysosomal exocytosis, which is an important process for cell homeostasis^18^. The polarity of cells is a key factor in intracellular trafficking and lysosomal exocytosis, and loss of hepatocyte polarity is observed in intra-/extra-hepatic cholestatic liver diseases^19,20^. In this study, cathepsin Z was increased in liver tissues from later stages of PBC via non-transcriptional regulation, and its expression in hepatocytes was correlated with bile duct loss. It is speculated that the loss of apical–basal polarity induces dysfunction of lysosome exocytosis and therefore dysfunction of biliary excretion as well, leading to increased expression and altered localization of lysosomal enzymes, including cathepsin Z, in hepatocytes of patients with PBC. Previously, UDCA was reported to insert MRP2 into canalicular membranes and stimulate organic anion secretion by protein kinase C–dependent mechanisms in a rat model of cholestasis^21^. However, in the current study, MRP2 was mainly localized in the cytoplasm of hepatocytes in jaundice-stage PBC and serum cathepsin Z levels did not decrease after UDCA treatment, indicating that UDCA is not effective at restoring hepatocyte polarity and excretion of cathepsin Z.

It has been reported that enlargement of the lysosome causes permeabilization of the lysosomal membrane and subsequent release of lysosomal enzymes into the cytoplasm, eventually leading to cell death^22^. Cathepsin Z was reported to be involved in the apoptosis of neurons^23^. In the current study, cathepsin Z was no longer colocalized with lysosomes in hepatocytes of jaundice-stage PBC patients, suggesting that cathepsin Z may be involved in hepatocyte cell death. Thus, increased expression and altered localization of cathepsin Z are not only a result of cholestasis, but may also be a cause of hepatocyte cell death, leading to the progression of PBC. This idea is supported by evidence that cathepsin Z polymorphisms are associated with the progression to jaundice stage in PBC^7^, although the functional role of the polymorphism is still under investigation. In addition, other cathepsin enzymes such as cathepsin B and D have been reported to be involved in cell death via lysosomal membrane permeabilization^22^. Further studies are needed to dissect the precise mechanism of cathepsin-induced hepatocyte cell death in PBC. Previously, the dysregulated maturation of cathepsin D was reported to be associated with pathogenesis of Alzheimer’s disease^24^. However, in this study, mature-form of cathepsin Z but not the pro-form was increased in the liver tissues from jaundice-stage PBC, suggesting that dysregulated maturation of cathepsin Z is not associated with the progression to jaundice stage in PBC.

In conclusion, we established that cathepsin Z is a useful marker for monitoring the progression of PBC. It is possible that inhibitors of cathepsin Z may attenuate cell death triggered by lysosomal membrane permeabilization and the subsequent mislocalization of cathepsin Z in PBC.

## Materials and Methods

The study subjects included 90 patients with PBC, 31 with chronic hepatitis C (CHC), 17 with obstructive jaundice, two with sepsis, two with Alagille syndrome, and six with no significant histopathological change (used as non-diseased liver), all of whom had been registered at the National Hospital Organization (NHO) Nagasaki Medical Center and Kanazawa University from December 1991 to July 2014. PBC patients were diagnosed based on established criteria^25^. CHC patients were diagnosed based on detection of hepatitis C virus RNA and anti-hepatitis C virus antibody in sera and were classified into early- and late-stage based on the Metavir scoring system, as described previously^26^. Patients with a score of 0–1 were classified as early-stage and patients with a score of 2–4 were classified as late-stage. PBC patients were classified into three clinical stages based on clinical manifestations as previously described^4^. The classification criteria were as follows: early-stage patients showed no signs of portal hypertension or cirrhosis; late-stage patients had signs of portal hypertension or cirrhosis, but not jaundice (total bilirubin <2 mg/dl); and jaundice-stage patients had persistent jaundice (total bilirubin ≥2 mg/dl). PBC patients with other concomitant liver diseases, including autoimmune hepatitis and viral hepatitis, were excluded from this study based on biochemical and histological findings.

Sera were obtained at the time of recruitment in this study. A part of sera from PBC patients who have undergone liver transplantation were collected before and after the transplantation. Liver tissues for subjects were collected at the time of diagnosis and/or during observation period. Demographics and clinical characteristics of PBC, CHC, and obstructive jaundice at the time of enrollment were shown in Table 1. Among of 90 PBC patients, 20 PBC patients underwent liver transplantation.

**Table 1:** Demographics and clinical characteristics of PBC, CHC, and obstructive jaundice patients at study enrollment.

	PBC (n = 90)	CHC (n = 31)	Obstructive jaundice (n = 17)
Age, mean ± SD (years)	59.3 ± 11.4	61.5 ± 9.6	70.9 ± 13.4
Female, n (%)	73 (81.1)	18 (58.1)	9 (52.9)
Early stage, n (%)	50 (55.6)	19 (61.3)	—
Late stage, n (%)	18 (20.0)	12 (38.7)	—
Jaundice stage, n (%)	22 (24.4)	—	—
ALT, mean ± SD	59 ± 41	59 ± 32	144 ± 135
ALP, mean ± SD	734 ± 576	324 ± 115	1414 ± 1086
T-Bil, mean ± SD	3.4 ± 6.3	1.1 ± 0.4	8.8 ± 8.1
No medication, n (%)	57 (63.3)	23 (74.2)	—
UDCA alone treatment^a^, n (%)	27 (30.0)	5 (16.1)	—
UDCA + bezafibrate treatment^b^,			
n (%)	6 (6.7)	0 (0)	—
Interferon treatment, n (%)	0 (0)	3 (9.7)	—

SD; standard deviation, ALT; alanine aminotransferase, ALP; alkaline phosphatase, T-Bil; total bilirubin, UDCA; ursodeoxycholic acid.

^a^dose of UDCA; 300−1200 mg/day.

^b^dose of bezafibrate; 200−400 mg/day.

### Immunohistochemistry and immunofluorescence staining

Hematoxylin and eosin staining was performed on 4-μm-thick, formalin-fixed, paraffin-embedded sections. Histological evaluation was performed as described elsewhere^27^. Immunohistochemistry was performed using a standardized two-step method with Histofine-Simple Stain (Nichirei Biosciences, Tokyo, Japan). Goat anti–human cathepsin Z polyclonal antibody (AF934, diluted 1:100, R&D Systems, Minneapolis, MN) was used as a primary antibody. HRP-conjugated rabbit anti–goat polymer (Simple Stain MAX PO (G); Nichirei Biosciences) was used as a secondary antibody. The reaction products were visualized using 3,3′-diaminobenzidine (Dako) and counterstained with Mayer’s hematoxylin (Dako). As a negative control, normal goat IgG (R&D Systems) was used as the primary antibody. The stained sections were evaluated by a blinded pathologist.

For immunofluorescence staining, blocking was performed with 3% bovine serum albumin in PBS for 30 minutes. Primary antibodies [goat anti–human cathepsin Z polyclonal antibody (AF934, diluted 1:100, R&D Systems), rabbit anti-MRP2 (ab187644, diluted 1:500, Abcam), and rabbit anti-LAMP1 polyclonal antibody (ab24170, diluted 1:250, Abcam)] were incubated overnight at 4 °C or for 1 hour at room temperature. Secondary antibody conjugated with Alexa 488 (diluted 1:300, Abcam) or Alexa 594 (diluted 1:500, Abcam) was incubated for 1 hour at room temperature. Nuclei were stained with VECTASHIELD Mounting Medium with DAPI (H-1200, Vector Laboratories, Burlingame, CA). Washing between each step was performed using 0.05% Tween-20 (Bio-Rad Laboratories, Hercules, CA) in PBS.

### SDS-PAGE and western blotting

Liver tissues were homogenized with Protein Elution/HEPES buffer (Apro Science, Naruto, Japan) supplemented with protease inhibitor cocktail (Nacalai Tesque, Kyoto, Japan), using the Cell/Tissue resin kit (Apro Science). Protein concentration was quantified using the BCA assay (Thermo Fisher Scientific). Twenty micrograms of denatured protein was electrophoresed on a 10% SDS-PAGE gel (Bio-Rad Laboratories) and transferred to a PVDF membrane (Merck Millipore, Billerica, MA). Rabbit anti–cathepsin Z (ab180580, Abcam) and rabbit anti-actin (A2066, Sigma-Aldrich) were used as primary antibodies. Donkey anti–rabbit IgG HRP–linked F(ab’)^2^ (NA9340V, GE Healthcare Japan, Hino, Japan) was used as a secondary antibody. Signal was detected with LAS 3000 (Fujifilm, Tokyo, Japan). Densitometric analysis was performed using Multi Gauge ver2.0 software (Fujifilm).

### RNA isolation, reverse transcription, and quantitative PCR

Total RNA was isolated from liver tissues using ISOGEN (Nippon Gene, Tokyo, Japan). cDNA was synthesized using ReverTra Ace® qPCR RT Master Mix with gDNA Remover (Toyobo, Osaka, Japan). Quantitative PCR was performed using SYBR Premix Ex Taq II (Takara Bio, Kusatsu, Japan) and a Light Cycler system (Roche, Basel, Switzerland). The following primers were used: human cathepsin Z, forward: 5′-GAATTCATGGGGTGAACCAT-3′, reverse: 5′-TCCTTATAGGTGCTGGTCACG-3′; GAPDH, forward: 5′-TGAACGGGAAGCTCACTGG-3′, reverse: 5′-TCCACCACCCTGTTGCTGTA-3′. All primers were synthesized by Sigma Genosys (Hokkaido, Japan).

### ELISA

Serum cathepsin Z was measured using a human cathepsin Z ELISA kit (Cloud-Clone Corp., Wuhan, China). In brief, standards (156 pg/ml – 10 ng/ml) or 1/4 diluted serum samples were added to wells pre-coated with mouse anti–human cathepsin Z antibody and incubated for 2 hours at 37 °C. After removing the standards and samples, biotin-conjugated rabbit anti–human cathepsin Z antibody was added and incubated for 1 hour at 37 °C. After washing three times, avidin-conjugated horseradish peroxidase (HRP) was added and incubated for 30 minutes at 37 °C. After washing five times, the wells were developed with 3,3’,5,5’-tetramethylbenzidine substrate solution for 20 minutes at 37 °C followed by addition of sulfuric acid solution. The absorbance was measured at 450 nm. The detection limit of cathepsin Z was 62 pg/ml. All samples were run in duplicate. Anti-mitochondrial, anti-gp210 and anti-centromere antibodies were measured as previously described^4^.

### Ethics board

This study was approved by the Ethics Board at the National Hospital Organization Nagasaki Medical Center and was conducted after obtaining informed consent from each subject for the use of sera and liver tissue samples. All experiments were performed in accordance with relevant guidelines and regulations.

### Data availability statement

All data generated or analyzed during this study are included in this published article.

## Electronic supplementary material

Supplementary information
