# Supplementary material for: Increased expression and altered localization of cathepsin Z are associated with progression to jaundice stage in primary biliary cholangitis
Source: Sci Rep. 2018 Aug 7;8:11808. doi: 10.1038/s41598-018-30146-w (PMC6081405; doi:10.1038/s41598-018-30146-w)
Supplement: Supplementary file 1 — Supplementary information [file 41598_2018_30146_MOESM1_ESM.docx]

Increased expression and altered localization of cathepsin Z are associated with progression to jaundice stage in primary biliary cholangitis

Yoshihiro Aiba^1^, Kenichi Harada^2^, Masahiro Ito^1,3^, Takashi Suematsu^4^, Shinichi Aishima^5^, Yuki Hitomi^6^, Nao Nishida^7^, Minae Kawashima^8^, Mitsuhisa Takatsuki^9^, Susumu Eguchi^9^, Shinji Shimoda^10^, Hitomi Nakamura^1^, Atsumasa Komori^1,3^, Seigo Abiru^1^, Shinya Nagaoka^1^, Kiyoshi Migita^1,3^, Hiroshi Yatsuhashi^1,3^, Katsushi Tokunaga^6^ and Minoru Nakamura^1,3,11^

1. Clinical Research Center, National Hospital Organization Nagasaki Medical Center, Omura, Japan.

2. Department of Human Pathology, Kanazawa University Graduate School of Medicine, Kanazawa, Japan.

3. Department of Hepatology, Nagasaki University Graduate School of Biomedical Sciences, Omura, Nagasaki, Japan.

4. Central Electron Microscope Laboratory, Nagasaki University School of Medicine, Nagasaki, Nagasaki, Japan.

5. Departments of Pathology & Microbiology, Faculty of Medicine, Saga University, Saga, Japan.

6. Department of Human Genetics, Graduate School of Medicine, The University of Tokyo, Tokyo, Japan.

7. The Research Center for Hepatitis and Immunology, National Center for Global Health and Medicine, Ichikawa, Chiba, Japan.

8. Japan Science and Technology Agency (JST), Tokyo, Japan.

9. Department of Surgery, Nagasaki University Graduate School of Biomedical Sciences, Nagasaki, Japan.

10. Department of Medicine and Biosystemic Science Graduate School of Medical Sciences, Kyushu University, Fukuoka, Fukuoka, Japan.

11. Headquarters of PBC Research in the National Hospital Organization Study Group for Liver Disease in Japan (NHOSLJ), Omura, Japan.


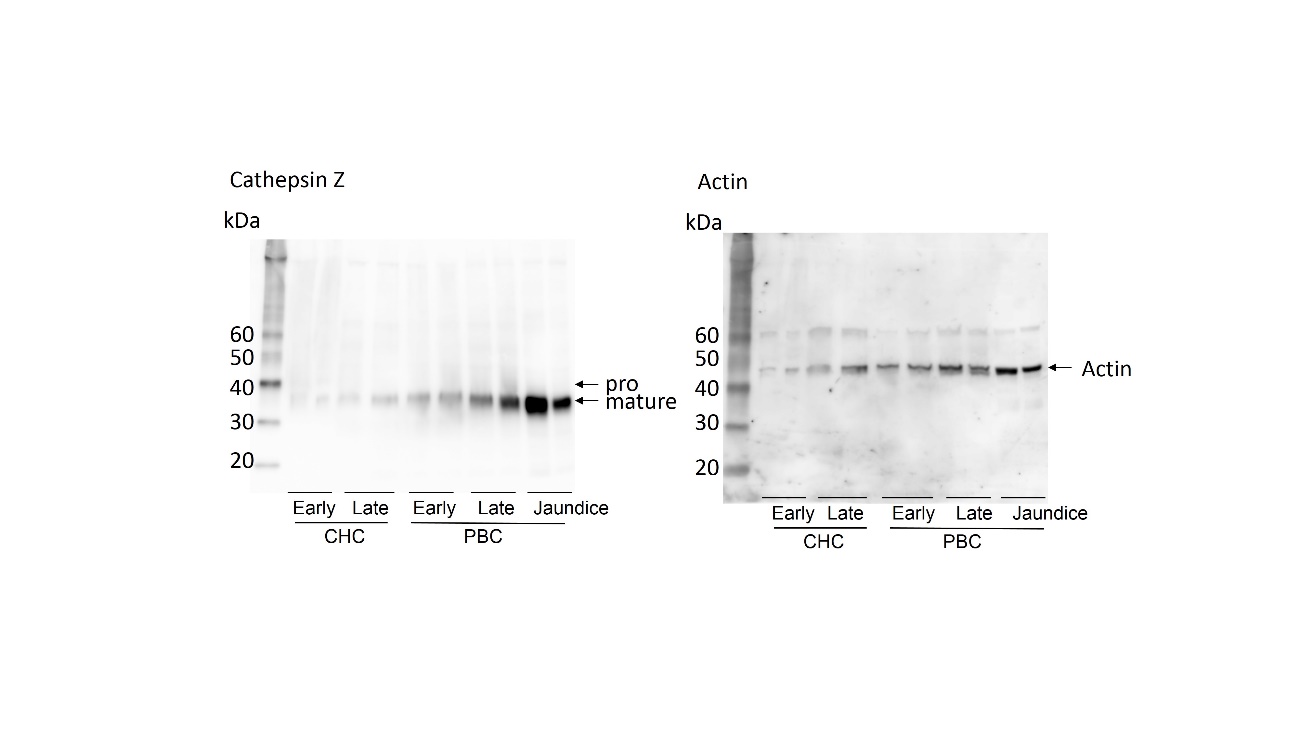


**Supplementary Figure 1.** Full-length blots of cathepsin Z and actin in liver tissues

Full-length blots for data shown in Figure 6 A.
